# Editorial: Therapeutic potential of adult neurogenesis in neurodegenerative and neuropsychiatric disorders

**DOI:** 10.3389/fnins.2025.1719276

**Published:** 2025-11-11

**Authors:** Ashok K. Shetty, Seiji Hitoshi

**Affiliations:** 1 Department of Cell Biology and Genetics, Institute for Regenerative Medicine, Texas A&M University Naresh K. Vashisht College of Medicine, Bryan/College Station, TX, United States; 2 Department of Integrative Physiology, Shiga University of Medical Science, Otsu, Shiga, Japan

**Keywords:** adult neurogenesis, cell grafting, dentate gyrus, hippocampus, lineage reprogramming, neural plasticity, neural repair, postnatal neurogenesis

Neurogenesis is the process by which new neurons are added to the brain from neural stem/progenitor cells (NSCs) (Bond et al., 2015; Elliott et al., 2025). While such process is highly apparent during brain development (Villalba et al., 2021), investigations over the last three decades have shown that neurogenesis continues into adulthood in specific brain areas, such as the hippocampus and the olfactory bulb (Bond et al., 2015; Salta et al., 2023; Elliott et al., 2025). Moreover, while the issue remains controversial, there is sufficient evidence from multiple studies to support neurogenesis occurring in the adult human hippocampus (Zanirati et al., 2023; Márquez-Valadez et al., 2025; Dumitru et al., 2025). Neurogenesis during brain development is a widespread process that builds the complex neural circuitry of the brain, occurring extensively across most regions (Villalba et al., 2021). In contrast, adult neurogenesis is limited to specific areas, particularly the hippocampus, producing new neurons for specific functions rather than overall brain expansion (Bond et al., 2015; Salta et al., 2023; Elliott et al., 2025).

Adult hippocampal neurogenesis (AHN) plays a vital role in maintaining hippocampus function and responding to new challenges (Bond et al., 2015; Salta et al., 2023; Elliott et al., 2025). Numerous studies have linked the extent of AHN to learning and memory, mood, cognitive flexibility, pattern separation, and brain plasticity (Bond et al., 2015; Elliott et al., 2025; Farmand et al., 2025). However, the factors such as sleep deprivation and stress can adversely influence the production and integration of these new neurons into existing hippocampal circuitry (Bond et al., 2015; Toda et al., 2019). The rate of adult hippocampal neurogenesis also decreases with age (Rao et al., 2006; Hattiangady and Shetty, 2008a,b; Hattiangady et al., 2008; Boldrini et al., 2018). In neurodegenerative conditions, such as Alzheimer's disease, it declines substantially due to various pathological factors, which may contribute to hippocampus-dependent cognitive impairments (Salta et al., 2023; Elliott et al., 2025). In contrast, in conditions such as status epilepticus, the rate of AHN increases dramatically, leading to abnormal migration of newly born neurons and the formation of dysfunctional circuitry, which likely contributes to chronic epilepsy (Rao et al., 2008). Waned neurogenesis has been linked to several neurological conditions, including the chronic phase of epilepsy and traumatic brain injury (Hattiangady and Shetty, 2008b; Kodali et al., 2023), and neuropsychiatric disorders (Hussaini et al., 2014; Márquez-Valadez et al., 2025). As a result, AHN is considered a potential therapeutic target for many neurological and neurodegenerative diseases. Since adult neurogenesis primarily occurs in the hippocampus in humans (Zanirati et al., 2023; Márquez-Valadez et al., 2025; Dumitru et al., 2025), emerging research is investigating the possibility of transforming non-neuronal cells in the brain into region-specific neurons by delivering reprogramming factors (Wei and Shetty, 2021; Bocchi et al., 2022; Marichal et al., 2024). This approach is still in its early stages, and its effectiveness is being studied across various models of neurological and neurodegenerative diseases (Peterson). If successful, it could revolutionize the replacement of specific types of neurons lost due to injury or disease.

This Research Topic collection comprises five original research articles and one review article, all published in Frontiers in Neuroscience. These studies, conducted on animal models, have provided new insights into the mechanisms underlying postnatal neurogenesis, adult neurogenesis, lineage programming, and stem cell grafting. The significant novel findings from these studies are summarized in the following section.

A study on neurogenesis during the development of the dentate gyrus (DG) reveals discrete transcriptional programs coordinating the differentiation and neurogenic progression of granule neuronal progenitors (GNPs) at embryonic vs. postnatal stages of DG neurogenesis (Ohyama et al.). Specifically, the study identified that during development, a sequential expression of the transcription factor Zeb1 is observed in neural stem progenitor pools, characterized by cells positive for GFAP and Sox2, followed by Scratch2 (Scrt2) expression in intermediate progenitors that are positive for Tbr2, Prox1, and NeuroD. Additionally, the study suggested that postnatal GNPs utilize the transcription factor Nkx6-2 to facilitate neuronal differentiation through epithelial-to-mesenchymal transition (EMT)-associated mechanisms (Ohyama et al.). A study by Miyamoto et al. provided insights into the gene expression profiles of neuroblasts migrating in the peri-injured cortex. They demonstrate that in neuroblasts migrating in the peri-injured cortex, the expression of genes involved in regulating migration direction and preventing cell death is upregulated, while the expression of genes involved in cell proliferation and maintenance of the immature state is downregulated. Additional analysis implied that in the injured brain, the proliferative activity of neuroblasts migrating toward lesions is suppressed by TGF-β secreted from microglia and macrophages surrounding the lesion (Miyamoto et al.). The results highlighted that migrating neuroblasts can exhibit slightly but distinctly different properties depending on the microenvironment along their journey.

In another study, Otsubo and associates employed an adeno-associated virus knockdown system in mice, providing evidence that Desmoplakin (Dsp), a component of desmosomal cell-cell junctions, has a role in maintaining DG function, including neuronal activity and adult neurogenesis, and anxiolytic-like effects (Otsubo et al.). Dsp expression was observed primarily in mature dentate granule cells, and its knockdown resulted in reduced expression of the activity-dependent transcription factor FosB, as well as increased expression of calbindin, a mature neuronal marker. Additionally, knockdown of Dsp in DG diminished the serotonin responsiveness of synapses formed by dentate granule cell axons, adversely affecting adult neurogenic processes in the DG and altering behavioral outcomes in a test for anxiety-like behavior. Overall, the study uncovered a previously unknown function of Dsp in the DG. However, it remains to be determined how Dsp binds to dentate granule cells and how it regulates neuronal activity, neurogenesis, and emotional behaviors.

Two additional articles in this Research Topic focus on the altered development of DG neurogenesis and its impact on epileptic susceptibility, as well as the role of seizure-induced neurogenesis in cognitive impairments. The study by Ruiz-Reig et al. investigated the functional consequences of p53 deletion in the cortex and hippocampus by generating a conditional mutant mouse (p53-cKO) in which p53 is deleted from pallial progenitors and their derivatives. Interestingly, such deletion did not alter the number of neurons in the cortex or the hippocampal cornu ammonis but led to increased proliferative cells in the subgranular zone of the DG and more granule cells in the granule cell layer of the DG. Additionally, p53-cKO mice exhibited a higher density of glutamatergic synapses in the CA3 region, resulting in hyperexcitability and increased epileptic susceptibility (Ruiz-Reig et al.). The authors suggest that, considering the role of p53 in the proliferation and self-renewal of neural stem cells in the subventricular zone, its potential role in glioblastoma genesis warrants further investigation. The study by Francis et al. investigated whether reducing the aberrant increase in neurogenesis could prevent cognitive impairments that emerge in the chronic epilepsy phase. In a long-term amygdala kindling model (consisting of 99 electrical stimulations), they showed that treatment with temozolomide, a DNA-alkylating agent, during a period of heightened neurogenesis can reduce aberrant neurogenesis in the hippocampus and prevent impairments in contextual fear discrimination and object recognition memory tasks (Francis et al.). Overall, the study implied that strategies that can selectively reduce aberrant adult neurogenesis could prevent cognitive deficits associated with chronic epilepsy.

In addition to the original research articles discussed above, the Research Topic collection includes a mini-review article that critically discusses the promise of recruiting resident non-neuronal cells by lineage programming, vis-à-vis replacing lost neurons via grafting of stem cell-derived neurons (Peterson). The review highlighted that the therapeutic recruitment strategy enables the more precise control of the location, connectivity, and extent of replacement neurons, thereby providing a wider range of therapeutic options than those offered by the engraftment of stem cell-derived neurons alone. However, the review noted that although progress has been made in recruiting resident non-neuronal cells using a direct *in vivo* reprogramming strategy, further refinements in efficiency and subtype specification are needed to advance this strategy. The review also highlighted the current limitations of the approach of direct reprogramming of non-neuronal cells, including the low conversion yield, preciseness in targeting only non-neuronal cells, the use of viral vectors for reprogramming, potential loss of cells because of unsuccessful reprogramming, yet to be proven long-term survival and integration of reprogrammed neurons and the need to test the efficacy of reprogramming strategies to convert human non-neuronal cells implanted into the brain in animal models into Bonafide mature neurons (Peterson).

In summary, the article collection in this Research Topic, representing the second volume of the RT “Advances in Adult Neurogenesis,” provides several novel insights into neurogenesis in the developing and adult DG, injured cortex, and aberrant neurogenesis in pathological conditions such as chronic epilepsy. In addition, the mini-review article weighs the pros and cons of *in vivo* reprogramming vs. stem cell-derived neuronal grafting for replacing lost neurons in the brain affected by neurological and neurodegenerative conditions.
